# Brachial-ankle pulse wave velocity predicts liver volume in patients with autosomal dominant polycystic kidney disease

**DOI:** 10.1371/journal.pone.0328133

**Published:** 2025-07-21

**Authors:** Wasako Tato, Tatsuya Suwabe, Yoshifumi Ubara, Yuki Oba, Hiroki Mizuno, Daisuke Ikuma, Masayuki Yamanouchi, Noriko Inoue, Akinari Sekine, Kiho Tanaka, Eiko Hasegawa, Takehiko Wada, Naoki Sawa

**Affiliations:** 1 Department of Nephrology, Toranomon Hospital Kajigaya, Kawasaki, Japan; 2 Department of Nephrology, Nara Medical University, Kashihara, Japan; 3 Okinaka Memorial Institute for Medical Research, Toranomon Hospital, Tokyo, Japan; 4 Department of Nephrology, Toranomon Hospital, Tokyo, Japan; Showa University, JAPAN

## Abstract

**Background:**

Autosomal dominant polycystic kidney disease (ADPKD) is the most common inherited kidney disease and Polycystic liver disease (PLD) is the most common extrarenal manifestation of ADPKD. Various non-inherited factors have been reported to affect total kidney volume (TKV) in ADPKD. However, the non-inherited factors affecting liver volume (LV) in ADPKD are unknown.

**Methods:**

We aimed to identify the factors affecting LV and TKV in ADPKD; and to analyze the relationship between changes in these parameters and arterial stiffness, assessed using brachial-ankle pulse wave velocity (baPWV).

**Results:**

We enrolled 165 patients (66 men and 99 women; mean age 47.3 ± 6.9 years). Univariable analysis revealed that sex, mean baPWV, ΔbaPWV, tolvaptan use, hyperlipidemia, hyperuricemia, Hb concentration, eGFR, proteinuria, and height-adjusted TKV (htTKV) were significantly associated with height-adjusted LV (htLV) at baseline. Multivariate analysis showed that sex, BMI, ΔbaPWV, and tolvaptan use were significantly associated with htLV at baseline. The baseline htLV correlated with ΔbaPWV (*r* = 0.32, *p* < 0.0001). Univariable linear mixed model analysis revealed that sex, mean baPWV, ΔbaPWV, tolvaptan use, hyperuricemia, Hb concentration, eGFR, and proteinuria significantly affected the change in htLV. Multivariate linear mixed model analysis revealed that sex, BMI, and tolvaptan use significantly affected the change in htLV. The change in the htLV ratio was larger in patients with a higher ΔbaPWV (*p* < 0.0001). Whereas, ΔbaPWV was not a significant factor for the baseline htTKV and the changes in htTKV in univariable and multivariable analysis.

**Conclusions:**

We have shown that ΔbaPWV is a predictor of baseline htLV, and the chronological changes in htLV in patients with ADPKD.

## Introduction

Autosomal dominant polycystic kidney disease (ADPKD) is the most common inherited kidney disease. ADPKD is characterized by the development and growth of cysts that cause enlargement and distortion of the kidneys and impair renal function. It is the fourth leading cause of end-stage kidney disease (ESKD) in adults worldwide [1–3]. Polycystic liver disease (PLD) is the most common extrarenal manifestation of ADPKD. Hepatic cysts are evident in 94% of patients with ADPKD who are > 35 years of age [4,5], and some patients experience severe enlargement of their polycystic liver.

ADPKD is an inherited disease, but various non-inherited factors have been reported to affect its prognosis. Rigorous blood pressure control is recommended for patients with early-stage ADPKD [6]; and salt intake, serum lipid concentration, smoking, and obesity also affect the prognosis of patients with ADPKD [7,8]. Many of these factors are known to be risk factors for arterial stiffness/elasticity, but the relationship between arterial stiffness itself and the progression of ADPKD has not been characterized. In addition, useful predictors of the progression of PLD have not been described in patients with ADPKD [9,10]. It has been reported that the severity of PLD is not markedly affected by genotype [9,10], which suggests that liver volume (LV) is more highly influenced by non-inherited factors than total kidney volume (TKV). Therefore, in the present study, we aimed to identify non-inherited factors that affect kidney and liver volume in ADPKD, and to characterize the relationship between arterial stiffness and the progression of PKD and PLD. We used pulse wave velocity (PWV) to evaluate arterial stiffness, because it is widely recognized to be a simple and reliable clinical measure of arterial stiffness/elasticity [11].

## Methods

We performed a retrospective, cohort study to investigate the relationship of PWV with LV and TKV in patients with ADPKD who were not on dialysis at Toranomon Hospital (Tokyo, Japan) or Toranomon Hospital Kajigaya (Kawasaki, Japan). The study was approved by the Ethics committee of Toranomon Hospital and Toranomon Hospital Kajigaya in November 2021 (approval number: 2282-B). Because the study was limited to a retrospective review of the imaging data and medical records of a subset of the enrolled patients, their specific informed consent was not required. We also used an opt-out approach that involved posting information regarding the study on the hospital’s website. The study was conducted according to the principles of the Declaration of Helsinki.

The clinical data in medical records were accessed for research purposes by a single group of research assistants from February 2022 to January 2023. The authors did not have access to information that could identify individual participants during data collection

### Participants

All the patients with ADPKD aged 20–60 years who were not on dialysis and underwent the measurement of PWV at Toranomon Hospital or Toranomon Hospital Kajigaya between January 2008 and January 2022 were enrolled. We recommended the measurement of brachial-ankle pulse wave velocity (baPWV) for patients with ADPKD to evaluate their arterial stiffness because many known prognostic factors for ADPKD are associated with arterial stiffness. The patients were adults (≥20 years old) who met the criteria for the diagnosis of ADPKD described by Pei *et al*. [12] and by the Progressive Renal Disease Research report of the Ministry of Health, Labour, and Welfare of Japan (S1 Table). The following participants were excluded from the study: those who did not undergo computed tomography (CT) within the 1.5 years before and after PWV measurement; those who did not undergo imaging on multiple occasions at ≥1 year intervals (n = 15); those who underwent baseline CT imaging following renal or liver drainage, resection, or hepatic-TAE (n = 50); those whose ankle-brachial index (ABI) was < 0.9 (n = 1), and those under 30 years old (n = 3). None of the participants had atrial fibrillation and one had undergone ablation as a treatment for atrial fibrillation, resulting in normal sinus rhythm at the time of PWV measurement. The participants were all Japanese, except for one from the Philippines.

### Clinical and laboratory assessments

We defined the time at which baPWV was measured as the baseline. The clinical features of the participants, including their height and weight, and medical history, were recorded at baseline. The blood pressure (BP) of the participants was measured using an automatic device while they were sitting. Body mass index (BMI) was calculated as body mass (kg) divided by the square of height (m). We defined hypertension as the use of antihypertensive drugs or a systolic BP > 140 mmHg, hyperlipidemia as the use of anti-hyperlipidemic drugs or a serum LDL-cholesterol concentration > 140 mg/dL, and hyperuricemia as the use of uric acid-lowering drugs or a serum uric acid concentration > 8 mg/dL at baseline. Laboratory testing was performed before baPWV was measured using automated standardized methods at our hospital within 24 h of the collection of the blood samples. Estimated glomerular filtration rate (eGFR) was used as a marker of renal function and was calculated using the simplified MDRD equation, modified by the appropriate coefficient for Japanese populations and according to sex, as follows: eGFR = 194 × Cr^−1.094^ × Age^−0.287^(×0.739 for women) (mL/min/1.73m^2^) [13].

### Imaging studies

CT was performed using a 16-MDCT scanner and 5-mm slices. Kidney and liver volumes were determined using Vincente software, version 4 (Fujifilm Co., Tokyo, Japan) by a single group of medical staff. TKV and LV were measured following automatic delineation and manual adjustment using this software. CT findings made within the year before or the year after PWV measurement were defined as the baseline findings. If the participants underwent renal or hepatic-TAE, or renal or hepatic cyst drainage, we did not review the subsequent imaging findings. We also censored participants when they underwent kidney or liver transplantation or initiated dialysis, because renal replacement therapy affects TKV and LV [14].

The height-adjusted LV (htLV) and htTKV ratios at each time point were calculated as follows: htLV (or htTKV) ratio = htLV (or htTKV) at each time point/ htLV (or htTKV) at baseline.

### Pulse wave velocity

PWV is widely recognized to be a simple and reliable clinical measure of arterial stiffness/elasticity, which is associated with vascular disease [11]. PWV is defined as the velocity with which the arterial blood pressure pulses propagate. baPWV measurement is commonly used in Japan because it can be performed by simply attaching blood pressure cuffs to all four extremities, rather than requiring the use of the femoral artery and exposure of the groin. In the present study, arterial stiffness was assessed by measuring baPWV and ABI using an automatic waveform analyzer (Form/ABI; Omron-Colin Co., Ltd., Komaki, Japan), in accordance with the manufacturer’s recommendations.

It has been reported that baPWV has prognostic significance when the presence of peripheral artery disease (PAD) is excluded using ABI, but the prognostic significance of baPWV disappears when PAD is not excluded [15]. Therefore, we excluded participants whose ABI was < 0.9 in the present study. baPWV is influenced by age and sex [16]; therefore, the arterial stiffness/elasticity of each patient was evaluated by comparing their baPWV value with those of healthy controls, which have been reported previously [16]. The healthy controls aged 30–60 years were considered to be suitable comparators; therefore, we enrolled patients aged 30–60 years in the present study. The differences in the baPWV of the participants and healthy controls were calculated using the following formula: ΔbaPWV = baPWV of each participant − the mean value for controls of the same age and sex. The healthy controls were individuals who were not on any medication and had no history of atherogenic disease, cardiovascular disease, renal insufficiency (serum creatinine concentration ≥ 1.5 mg/dL), hypertension, hyperlipidemia, high uric acid concentration, obesity, smoking, or any other diseases requiring medical treatment [16]. The mean values for parameters for the healthy controls are used as references.

### Statistical analysis

The clinical features and laboratory data for the participants at baseline were analyzed. Normally distributed continuous data are presented as mean ± SD, and non-normally distributed numeric continuous data are presented as median (interquartile range). Univariable and multivariable regression models were used to identify factors affecting htLV and htTKV at baseline, and predictors were chosen in the multivariable analysis using the stepwise selection method. The regression coefficients (95% CIs) for the relationships of the htLV and htTKV ratios with baseline variables were calculated using univariable linear mixed models. In addition, the changes in the htLV and htTKV ratios over 1 year, estimated using linear mixed effects models, were analyzed as the response variables. The baseline variables were age, sex, BMI, systolic BP, diastolic BP, heart rate, mean baPWV (mean of the left and right baPWV), ΔbaPWV, Hb, eGFR, urine protein concentration, htLV, htTKV, history of smoking, use of tolvaptan, and previous medical history (cardiovascular disease, cerebrovascular disease, cerebral aneurysm, subarachnoid hemorrhage, sleep apnea syndrome, malignant neoplasm, diabetes mellitus, hypertension, hyperlipidemia, hyperuricemia, and renal or liver cyst infection). Because the data were skewed, logarithmic transformation was performed for proteinuria, htLV, and htTKV. Potential confounding variables and interactions were also evaluated. Curves of the mean htLV and htTKV ratios (95% CIs) against time were constructed using generalized additive models. To graphically evaluate the effects of ΔbaPWV on the changes in the htLV and htTKV ratios, quartiles of ΔbaPWV were used and the curves of the mean values *vs*. time point were constructed using generalized additive models. To graphically evaluate the relationships between htLV and ΔbaPWV, htTKV and ΔbaPWV, and htTKV and eGFR, scatter plots and regression lines were created. Logarithmic transformation was performed for the htLV, and htTKV data used in the scatter plot.

Analyses were performed using SAS, version 9.3 (SAS Institute Inc., Cary, NC, USA), and *p* < 0.05 was considered to indicate statistical significance.

## Results

### Study population

**Fig 1:**
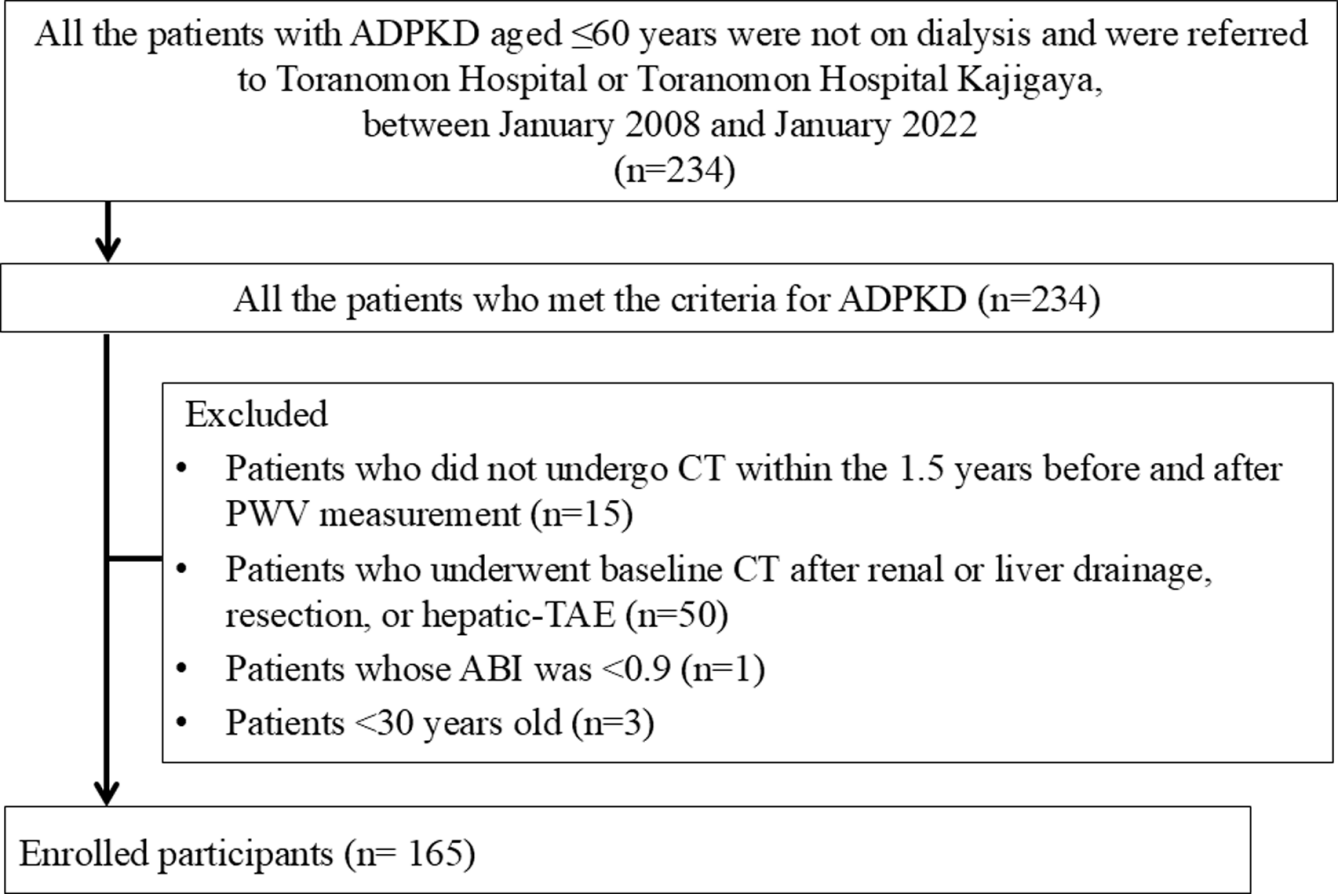
Flowchart showing the enrollment of all the patients with ADPKD who were aged ≤60 years, were not undergoing dialysis, and were referred to Toranomon Hospital, Kajigaya, between January 2008 and October 2021. Abbreviations: ADPKD, autosomal dominant polycystic kidney disease.

**Fig 2:**
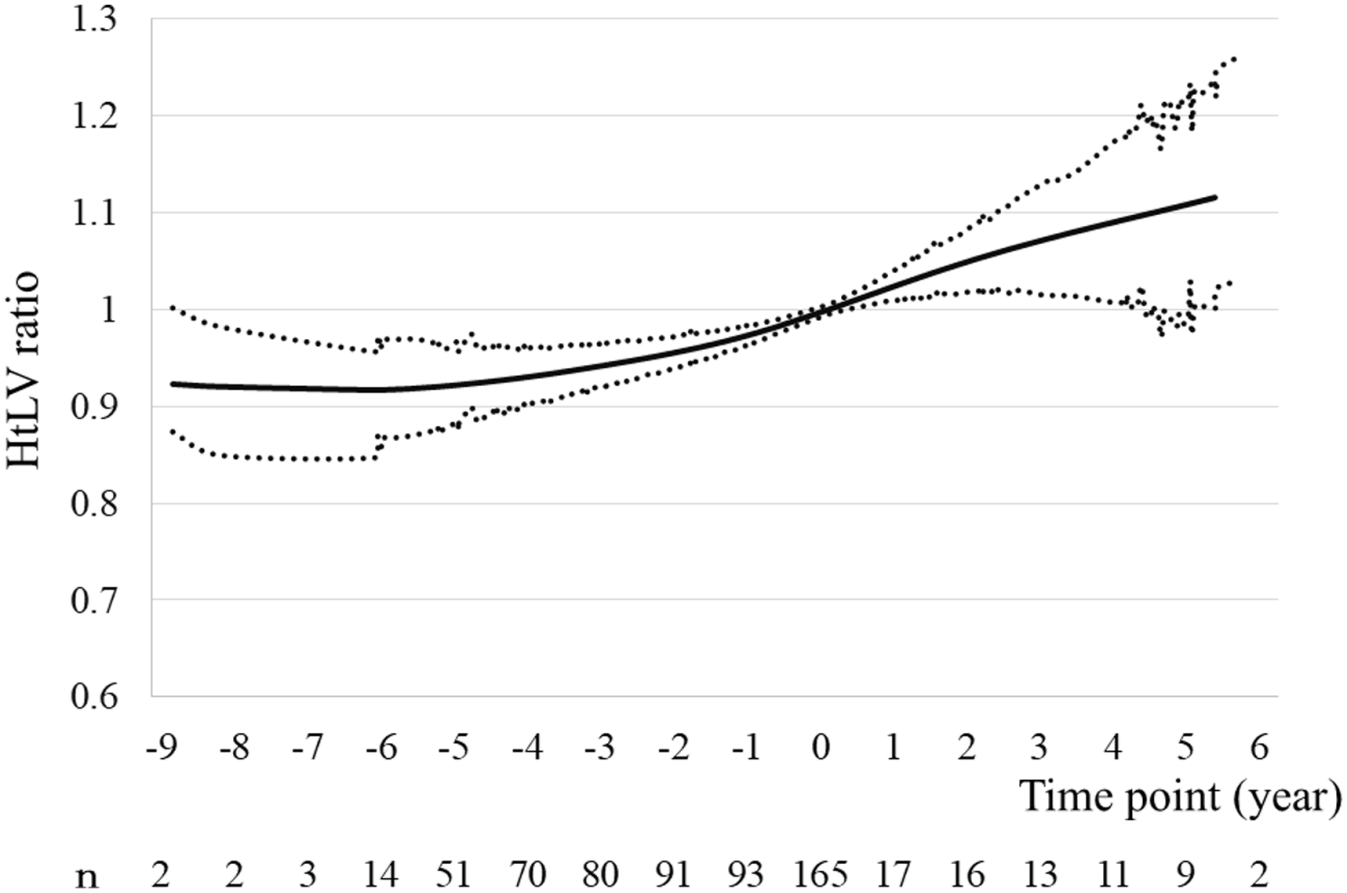
Changes in the mean htLV ratios with time. Graph of the mean htLV ratio (95% confidence interval) against time, created using a generalized additive model. Abbreviations: htLV, height-adjusted liver volume.

**Fig 3:**
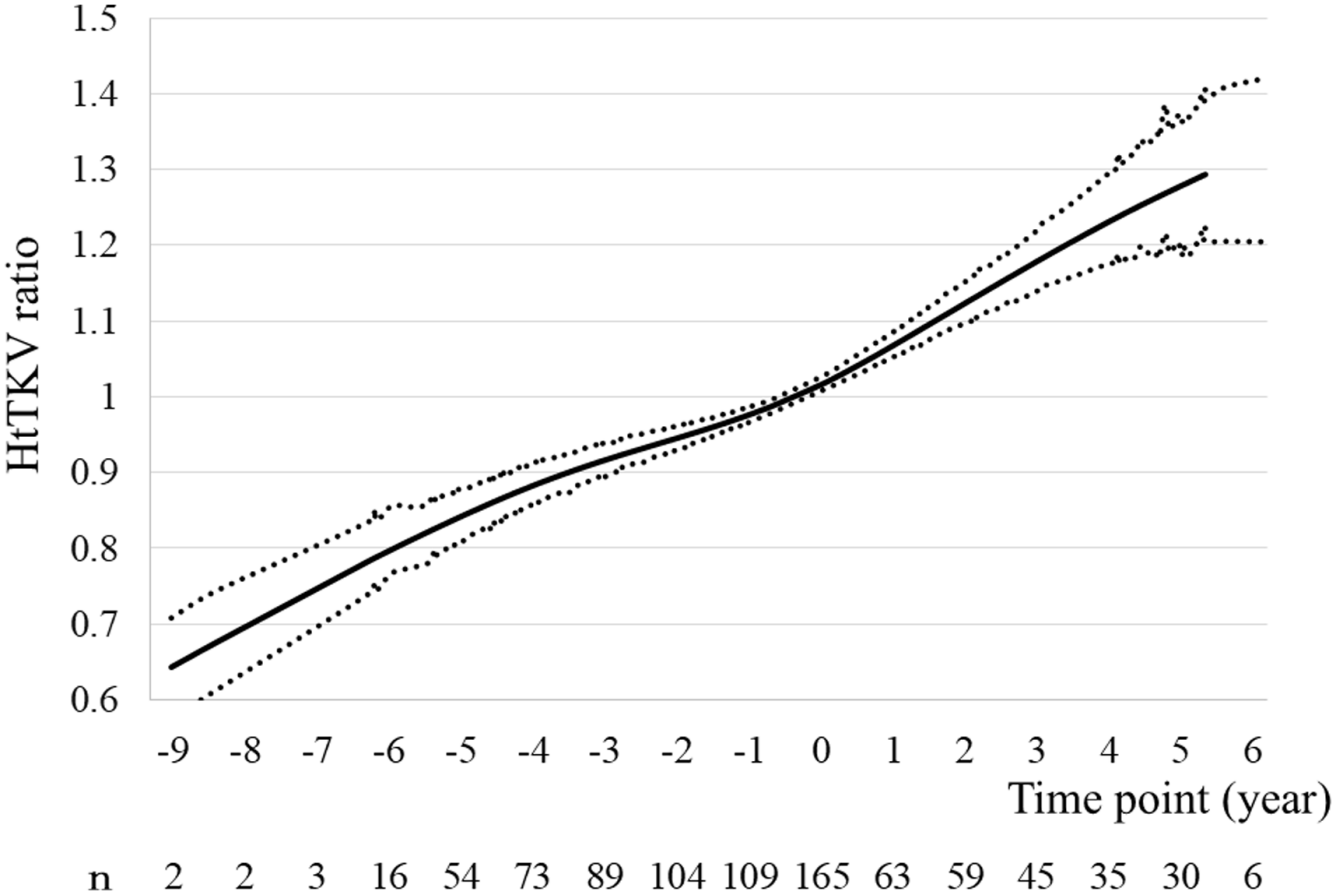
Changes in the mean htTKV ratios with time. Graph of the mean htTKV ratio (95% confidence interval) against time, created using a generalized additive model. Abbreviations: htTKV, height-adjusted total kidney volume.

A total of 234 patients with ADPKD who were aged ≤60 years, were not undergoing dialysis, and were referred to Toranomon Hospital or Toranomon Hospital Kajigaya, underwent baPWV measurement between January 2008 and January 2022. Of these, 69 were excluded (Fig 1). The remaining 165 comprised 66 men and 99 women with a mean age of 47.3 ± 6.9 years (Table 1). Two patients underwent kidney or liver transplantation, and 10 patients initiated dialysis during the study period. Their median (interquartile range) TLV was 2,551 ml (1,598–6,018 ml), their mean baPWV was 1,430.0 ± 233.7, and their mean ΔbaPWV was 253.3 ± 230.0. The changes in the htLV, and htTKV ratios at each time point are presented in Fig 2, 3.

**Table 1:** Clinical characteristics of all enrolled patients.

	All patients	Quartile 1^a^ (n = 42)	Quartile 2^b^ (n = 41)	Quartile 3^c^ (n = 41)	Quartile 4^d^ (n = 41)	^e^ P value
Number (M/F)	165 (66/ 99)	42 (30/ 12)	41 (27/ 14)	41 (22/ 19)	41 (20/ 21)	0.1237
Age (years) [mean±SD]	47.3 ± 6.9	47.2 ± 6.9	47.9 ± 7.7	47.0 ± 6.2	47.1 ± 7.1	0.9378
Height (cm) [mean±SD]	165.9 ± 8.8	163.4 ± 7.6	165.2 ± 8.1	166.1 ± 9.2	169.1 ± 9.8	0.0286
Body weight (kg) [mean±SD]	64.2 ± 15.1	59.6 ± 11.6	68.0 ± 17.5	62.6 ± 14.2	67.1 ± 15.5	0.0366
BMI [mean±SD]	23.1 ± 3.9	22.2 ± 3.4	24.7 ± 4.7	22.4 ± 3.3	23.3 ± 4.1	0.0191
Systolic blood pressure (mmHg) [mean±SD]	126.0 ± 15.9	133.3 ± 16.8	130.8 ± 14.4	120.3 ± 12.6	116.7 ± 13.2	<0.0001
Diastolic blood pressure (mmHg) [mean±SD]	81.7 ± 12.0	86.8 ± 11.8	84.4 ± 10.0	76.9 ± 11.5	76.8 ± 11.7	0.0008
Heart rate (per min) [mean±SD]	70.2 ± 11.9	73.4 ± 13.7	73.3 ± 14.1	67.3 ± 7.9	65.8 ± 8.8	0.0521
Right ABI [mean±SD]	1.14 ± 0.07	1.15 ± 0.07	1.15 ± 0.08	1.14 ± 0.07	1.14 ± 0.08	0.7201
Left ABI [mean±SD]	1.13 ± 0.07	1.15 ± 0.07	1.15 ± 0.07	1.13 ± 0.07	1.13 ± 0.08	0.4329
Right baPWV [mean±SD]	1421.2 ± 225.7	1687.3 ± 169.6	1487.7 ± 98.4	1340.8 ± 80.1	1162.9 ± 97.1	<0.0001
Left baPWV [mean±SD]	1438.8 ± 244.7	1738.2 ± 184.7	1505.5 ± 108.2	1343.2 ± 63.3	1161.0 ± 88.0	<0.0001
Mean baPWV [mean±SD]	1430.0 ± 233.7	1712.7 ± 174.2	1496.6 ± 100.4	1342.0 ± 67.4	1162.0 ± 90.6	<0.0001
ΔbaPWV [mean±SD]	253.3 ± 230	548.7 ± 154.0	317.7 ± 50.5	163.4 ± 41.7	−23.7 ± 75.3	<0.0001
Smoking history [n, (%)]	47 (28.5%)	7 (16.7%)	12 (29.3%)	13 (31.7%)	15 (36.6%)	0.1504
Medications						
Tolvaptan	88 (53.3%)	16 (38.1%)	24 (58.5%)	24 (58.5%)	24 (58.5%)	0.1529
Past medical history						
Cardiovascular disease	7 (4.2%)	3 (7.1%)	0 (0%)	3 (7.3%)	1 (2.4%)	0.1412
Cerebral vascular disease	5 (3.0%)	1 (2.4%)	1 (2.4%)	2 (4.9%)	1 (2.5%)	0.9026
Cerebral aneurysm	18 (10.9%)	5 (11.9%)	7 (17%)	3 (7.3%)	3 (7.3%)	0.4484
Subarachnoid hemorrhage	6 (3.6%)	3 (7.1%)	2 (4.9%)	0 (0%)	1 (2.4%)	0.2079
Sleep Apnea Syndrome	8 (4.8%)	0 (0%)	5 (12.2%)	2 (4.9%)	1 (2.4%)	0.0413
Malignant neoplasm	5 (3.0%)	4 (9.5%)	0 (0%)	0 (0%)	1 (2.4%)	0.0294
Diabetes mellitus	3 (1.8%)	0 (0%)	2 (4.9%)	0 (0%)	1 (2.4%)	0.2032
Hypertension	125 (75.8%)	31 (73.8%)	36 (87.9%)	28 (68.3%)	30 (73.1%)	0.1607
Hyperlipidemia	25 (15.1%)	4 (9.5%)	11 (26.8%)	5 (12.2%)	5 (12.2%)	0.1421
Hyperuricemia	58 (35.1%)	10 (23.8%)	14 (34.2%)	17 (41.5%)	18 (43.9%)	0.2079
Renal or Liver cyst infection	9 (5.4%)	4 (9.5%)	6 (14.6%)	3 (7.3%)	2 (4.8%)	0.4703
Laboratory data						
Hb (g/dL) [mean±SD]	12.9 ± 1.5	13.0 ± 1.8	12.7 ± 1.7	12.8 ± 1.2	13.0 ± 1.4	0.8768
Cr (mg/dL) [median (IQR)]	1.2 (0.8, 1.9)	1.2 (0.8, 2.2)	1.3 (0.8, 2.6)	1.2 (0.9, 1.7)	1.3 (0.8, 1.8)	0.8620
eGFR (ml/min/1.73m^2^) [mean±SD]	46.1 ± 24.0	45.2 ± 25.8	42.9 ± 25.3	46.9 ± 21.8	49.3 ± 23.2	0.6684
Proteinuria (g/gCr) [median (IQR)]	0.13 (0.06, 0.25)	0.15 (0.06, 0.30)	0.18 (0.09, 0.51)	0.10 (0.06, 0.18)	0.08 (0.05, 0.18)	0.0054
Image findings						
TKV (mL) [median (IQR)]	1329 (860, 2110)	1371 (958, 1736)	1500 (941, 2929)	1329 (865, 2031)	1013 (746, 2392)	0.5125
LV (mL) [median (IQR)]	2551 (1598, 6018)	5687 (2219, 8168)	2081 (1491, 6405)	2340 (1562, 4055)	1942 (1457, 3881)	0.0006
Observational periods (months) [median (IQR)]	48.2 (28.5, 55.9)	47.1 (25.3, 56.7)	49.6 (36.7, 58.0)	46.5 (28.3, 52.0)	48.4 (30.9, 54.0)	0.7488

SD, standard deviation; IQR, interquartile range (25% − 75%); BMI, body mass index; ABI, ankle brachial pressure Index; baPWV, brachial-ankle pulse wave velocity; ΔbaPWV, baPWV of each participant – the mean value for controls of the same age and sex; eGFR, estimated glomerular filtration rate; TKV, total kidney volume; LV, liver volume

^a^ Range for quartile 1: ΔPWV >=411.5

^b^ Range for quartile 2: ΔPWV >=236.5, < 411.5

^c^ Range for quartile 3: ΔPWV >=100, < 236.5

^d^ Range for quartile 4: ΔPWV <100

^e^ Differences between four groups were assessed by analysis of variance for continuous variables with a normal distribution and the Kruskal-Wallis test for continuous variables with a non-normal distribution. Differences of categorical variables between groups were assessed by the χ^2^ test or Fisher’s exact test.

The number of patients studied at Toranomon Hospital Kajigaya was twice the number at Toranomon Hospital (S2 Table). The patients at Toranomon Hospital Kajigaya had larger htLV and more patients at Toranomon Hospital were taking tolvaptan than at Toranomon Hospital Kajigaya. Hepatic-TAE was used for the assessment of an enlarged liver in patients with ADPKD only at Toranomon Hospital Kajigaya [17]; therefore, more patients with very large livers attended Toranomon Hospital Kajigaya.

### Predictive variables of htLV, and htTKV at baseline

**Fig 4:**
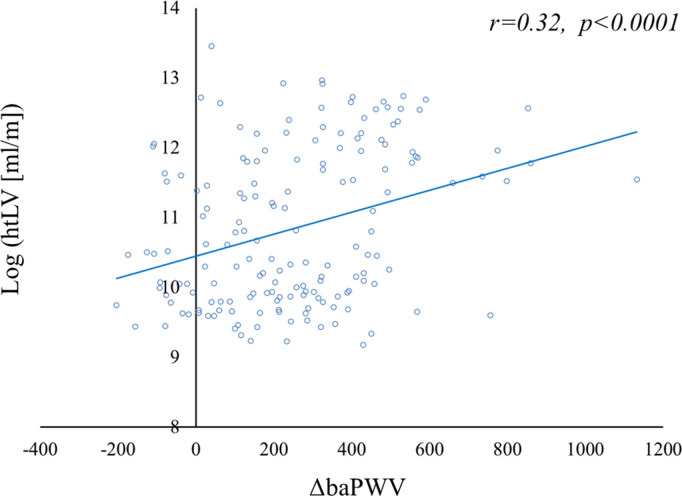
Correlation between log (htLV) and ΔbaPWV at baseline. The log (htLV) was significantly larger in participants with higher ΔbaPWV (*r* = 0.32, *p* < 0.0001). Abbreviations: htLV, height-adjusted liver volume; baPWV, brachial-ankle pulse wave velocity; ΔbaPWV, baPWV of each participant − the mean value for controls of the same age and sex.

Univariable analysis revealed that sex, mean baPWV, ΔbaPWV, tolvaptan use, hyperlipidemia, hyperuricemia, Hb concentration, eGFR, proteinuria, and htTKV were significantly associated with htLV (Table 2). ΔbaPWV correlated with log(htLV) (*r* = 0.32, *p* < 0.0001) (Fig 4). Multivariable analysis confirmed that sex, BMI, ΔbaPWV, and tolvaptan use were significantly associated with htLV (Table 2).

**Table 2:** Univariable and multivariable regression coefficient for height-adjusted liver volume in patients with ADPKD at baseline.

	Univariable analysis				Multivariable analysis ^a^			
	Regression coefficient	95% CI		P value	Regression coefficient	95% CI		P value
Sex (Male)	−0.5692	−0.7955	−0.3429	<.0001	−0.6654	−0.9707	−0.3602	<.0001
Age (per 1 year)	−0.0044	−0.0216	0.0129	0.6184				
BMI (per 1)	0.0226	−0.0072	0.0525	0.1363	0.0714	0.0349	0.1079	0.0002
Systolic BP (per 1 mmHg)	0.0041	−0.0049	0.0131	0.3658				
Diastolic BP (per 1 mmHg)	0.0020	−0.0100	0.0139	0.745				
Heart rate (per 1)	0.0059	−0.0070	0.0187	0.3648				
Mean baPWV (per 1)	0.0007	0.0003	0.0012	0.0034				
ΔbaPWV (per 1)	0.0011	0.0006	0.0016	<.0001	0.0010	0.0004	0.0016	0.0010
Smoking history	−0.1526	−0.4187	0.1135	0.2591				
Tolvaptan	−0.7843	−0.9896	−0.5790	<.0001	−0.9063	−1.1828	−0.6298	<.0001
Cardiovascular disease	0.0559	−0.5342	0.6461	0.8518				
Cerebral vascular disease	−0.3482	−1.0400	0.3437	0.3219				
Cerebral aneurysm	−0.1011	−0.4824	0.2801	0.6011				
Subarachnoid hemorrhage	−0.0043	−0.6398	0.6312	0.9894				
Sleep Apnea Syndrome	−0.4332	−0.9830	0.1166	0.1217				
Malignant neoplasm	0.4711	−0.2190	1.1613	0.1795				
Diabetes mellitus	−0.6547	−1.5392	0.2299	0.1458	−0.7319	−1.7530	0.2891	0.1588
Hypertension	−0.1526	−0.4292	0.1240	0.2776				
Hyperlipidemia	−0.3290	−0.6569	−0.0012	0.0492				
Hyperuricemia	−0.3706	−0.6121	−0.1291	0.0028				
Renal or Liver cyst infection	−0.0778	−0.2087	0.0531	0.2422				
Hb (per 1 g/dL)	−0.1326	−0.2075	−0.0578	0.0006				
eGFR (per 1 ml/min/1.73m^2^)	0.0073	0.0025	0.0122	0.0033				
Log (Proteinuria [g/gCr])	0.1168	0.0159	0.2176	0.0235				
Log (htTKV[mL])	−0.2124	−0.3843	−0.0406	0.0157				

BMI, body mass index; baPWV, brachial-ankle pulse wave velocity; ΔbaPWV, baPWV of each participant – the mean value for controls of the same age and sex; eGFR, estimated glomerular filtration rate; htTKV, height-adjusted total kidney volume

^a^ These variables were selected by stepwise elimination.

**Fig 5:**
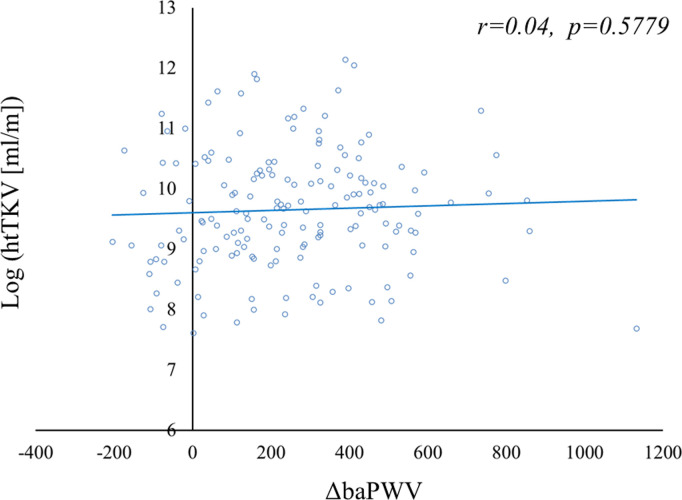
Correlation between log (htTKV) and ΔbaPWV at baseline. The log (htTKV) did not correlate with ΔbaPWV (*r* = 0.04, *p = *0.5779). Abbreviations: htTKV, height-adjusted total kidney volume; baPWV, brachial-ankle pulse wave velocity; ΔbaPWV, baPWV of each participant − the mean value for controls of the same age and sex.

Univariable analysis revealed that sex, BMI, systolic BP, diastolic BP, tolvaptan use, hypertension, hyperuricemia, eGFR, proteinuria, and htLV were significantly associated with htTKV (S3 Table). ΔbaPWV did not significantly correlate with log(htTKV) (*r* = 0.04, *p* = 0.5779) (Fig 5). Multivariable analysis showed that sex, age, eGFR, and proteinuria were significantly associated with htTKV (S3 Table).

### Predictive variables of sequential changes in htLV, and htTKV

**Fig 6:**
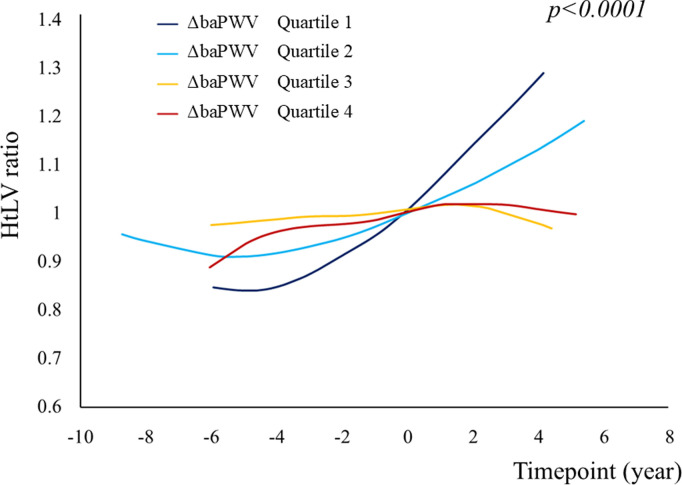
Graph of the mean htLV ratio against time, created using the generalized additive model, and stratified according to ΔbaPWV quartile. The change in the htLV ratio at each time point, stratified according to ΔbaPWV quartile, was larger in participants with higher ΔbaPWV (*p < *0.0001). The participants were grouped by ΔPWV as follows: quartile 1, ≥411.5; quartile 2, ≥236.5, <411.5; quartile 3, ≥100, <236.5; quartile 4, <100. Abbreviations: htLV, height-adjusted liver volume; baPWV, brachial-ankle pulse wave velocity; ΔbaPWV, baPWV of each participant − the mean value for controls of the same age and sex.

Univariable linear mixed model analysis revealed that sex, mean baPWV, ΔbaPWV, tolvaptan use, hyperuricemia, Hb concentration, eGFR, and proteinuria significantly affected the change in htLV (Table 3). Multivariable linear mixed model analysis revealed that sex, BMI, and tolvaptan use significantly affected the change in htLV (Table 3). The changes in the htLV ratio between each time point, stratified according to ΔbaPWV quartile, were larger in patients with a higher ΔbaPWV (*p* < 0.0001) (Fig 6).

**Table 3:** The changes (95% CIs) of slope coefficients of height-adjusted liver volume curves by predictive variables in univariable and multivariable linear mixed model analyses.

	Univariable analysis				Multivariable analysis ^a^			
	Regression coefficient	95% CI		P value	Regression coefficient	95% CI		P value
Sex (Male)	−1380.56	−1982.73	−778.39	<.0001	−1478.56	−2059.08	−898.04	<.0001
Age (per 1 year)	−16.01	−61.30	29.29	0.4863				
BMI (per 1)	92.78	15.02	170.53	0.0197	160.74	91.03	230.45	<.0001
Systolic BP (per 1 mmHg)	16.69	−8.33	41.72	0.1890				
Diastolic BP (per 1 mmHg)	16.42	−16.77	49.62	0.3290				
Heart rate (per 1)	33.11	−3.47	69.70	0.0755				
Mean baPWV (per 1)	1.75	0.43	3.06	0.0095				
ΔbaPWV (per 1)	2.61	1.31	3.92	0.0001				
Smoking history	−529.76	−1227.77	168.24	0.1359				
Tolvaptan	−1742.44	−2309.01	−1175.87	<.0001	−1422.17	−1957.11	−887.23	<.0001
Cardiovascular disease	−78.02	−1630.89	1474.86	0.9211				
Cerebral vascular disease	−647.00	−2470.18	1176.19	0.4845				
Cerebral aneurysm	−129.75	−1133.55	874.05	0.7989				
Subarachnoid hemorrhage	−9.00	−1681.07	1663.07	0.9915				
Sleep Apnea Syndrome	−1123.36	−2570.21	323.50	0.1272				
Malignant neoplasm	1276.47	−538.75	3091.69	0.1669				
Diabetes mellitus	−1552.36	−3882.69	777.97	0.1902				
Hypertension	−180.61	−910.44	549.23	0.6257				
Hyperlipidemia	−797.90	−1662.09	66.29	0.0701				
Hyperuricemia	−698.21	−1342.27	−54.14	0.0338				
Renal or Liver cyst infection	−159.96	−504.88	184.96	0.3612				
Hb (per 1 g/dL)	−391.03	−586.07	−196.00	0.0001				
eGFR (per 1 ml/min/1.73m^2^)	18.19	5.41	30.97	0.0055				
Log (Proteinuria [g/gCr])	195.39	11.03	379.75	0.0379				
Log (htTKV[mL])	−236.20	−553.23	80.83	0.1432				

BMI, body mass index; baPWV, brachial-ankle pulse wave velocity; ΔbaPWV, baPWV of each participant – the mean value for controls of the same age and sex; eGFR, estimated glomerular filtration rate; htTKV, height-adjusted total kidney volume

^a^ These variables were selected by stepwise elimination.

**Fig 7:**
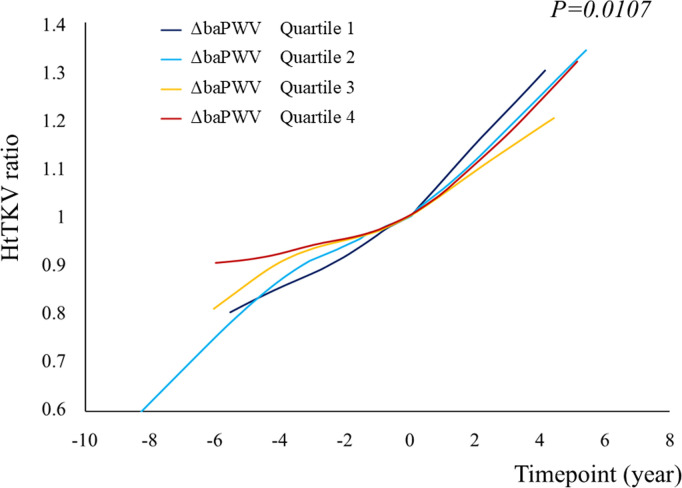
Graph of the mean htTKV ratio against time, created using a generalized additive model, and stratified according to ΔbaPWV quartile. The change in the htTKV ratio at each time point, stratified according to ΔbaPWV quartile, was larger in participants with higher ΔbaPWV (*p = *0.0107). The participants were grouped by ΔPWV as follows: quartile 1, ≥411.5; quartile 2, ≥236.5, <411.5; quartile 3, ≥100, <236.5; quartile 4, <100. Abbreviations: htTKV, height-adjusted total kidney volume; baPWV, brachial-ankle pulse wave velocity; ΔbaPWV, baPWV of each participant − the mean value for controls of the same age and sex.

Univariable linear mixed model analysis revealed that sex, BMI, systolic BP, diastolic BP, a history of smoking, tolvaptan use, hypertension, hyperuricemia, renal or liver cyst infection, eGFR, and proteinuria significantly affected the change in htTKV (S4 Table). Multivariable linear mixed model analysis revealed that sex, diastolic BP, eGFR, and proteinuria significantly affected the change in htTKV (S4 Table). The changes in the htTKV ratio between each time point, stratified according to ΔbaPWV quartile, were larger in patients with higher ΔbaPWV (*p* = 0.0107); however, the differences were small (Fig 7).

We conducted a subgroup analysis according to the age, sex, and LV of the participants. This revealed that LV was more closely associated with ΔbaPWV in older female patients with larger LV (S5, S6, S7 Tables). However, there were relatively few patients in each of the groups, so this analysis should be repeated in the future using a larger number of patients.

## Discussion

In the present study, we identified factors that influence baseline htLV, htTKV, and the longitudinal changes in these parameters. The factors found to affect the baseline htTKV and changes in htTKV comprised age, sex, eGFR, and proteinuria, which are well-known prognostic factors for TKV. However, prognostic biomarkers of LV have not previously been identified. In the present study, baPWV was found to be associated with both single values and the changes in htLV. ΔbaPWV was not a significant factor for change in LV in multivariable linear mixed model analysis using stepwise selection method in the whole patients, possibly because the effectiveness was not so strong, and the number of the observations was not enough to detect the significance.

The measurement of PWV is easy, cheap, and noninvasive, and the required equipment is widely available around the world. Therefore, this finding should have a significant impact on the management of patients with ADPKD.

PWV is regarded as the gold standard measure of arterial stiffness [11]. Carotid-femoral pulse wave velocity (cfPWV) has been validated and is widely used in clinical settings throughout Europe [18], and baPWV has been validated and is widely used in East Asian countries [19]. Close correlations have been reported between baPWV and cfPWV [20]. High baPWV values are typical in older people [16,20]; in patients with hypertension [16], diabetes [21], and metabolic syndrome [22]; in smokers [23]; in those who experience poor quality sleep [24]; and in those with high sodium intake [25]. baPWV has been shown to be predictive of cardiovascular events [26,27] and mortality [28] in a number of populations; and a high baPWV is associated with chronic kidney disease and proteinuria [29]. It has also been reported that PWV is associated with volume overload in patients with chronic kidney disease (CKD) who are not undergoing dialysis and in those undergoing hemodialysis or peritoneal dialysis [30–32].

Hypertension is a cause of CKD and it also contributes to its progression [33–35]. Salt intake is associated with hypertension and renal damage, as well as the growth of renal cysts, through the activation of vasopressin 2 receptor secondary to an increase in plasma osmolality in patients with ADPKD [36]. Obesity is also associated with renal damage, as well as the growth of renal cysts, through the activation of AMP-activated-protein kinase [7]. PWV is affected by all these factors; therefore, it is reasonable to suppose that both PWV and ΔPWV are related to TKV in patients with ADPKD. However, they did not significantly affect TKV in the present study. One of the reasons for this is that TKV was more substantially affected by renal function and was more closely related to renal function in the present study (S1, S2 Fig.). This finding is consistent with the consensus that TKV is a strong prognostic marker of renal function in ADPKD [37]. In addition, as we have previously reported, uremia itself may accelerate the increase in TKV, the decline in renal function, and the growth of TKV synergistically [14]. TKV may also be more affected by inherited factors than LV.

We have previously reported that the LV continues to increase, even after the initiation of dialysis, although the TKV decreases. However, the change in LV significantly decreases after dialysis commences, and the increases in TKV and LV are larger in patients undergoing PD than in those undergoing HD [14]. These results suggest that LV is influenced by hypertension or volume overload, because these are more common in patients undergoing PD [38]. We have also reported that rigorous BP control and an amelioration of interstitial tissue edema might reduce LV [39]. Therefore, we had expected that LV would be related to PWV to some extent, but surprisingly, LV more closely correlated with PWV than TKV in the present study. One reason for this was that the mean LV of the patients was much higher than normal in the present study, which may imply that more patients who were at risk of PLD progression were included.

The renal arteries originate from the abdominal aorta, whereas the hepatic artery is a branch of the celiac artery, and the pressure in the hepatic artery may be regulated to maintain this at a constant level [40]. Thus, PLD may be less affected by hypertension. However, PLD may be more affected by volume overload, because progression of PKD may be slowed by substantial water intake *via* a reduction in vasopressin. Body fluid overload may promote fluid movement to the interstitial space, as well as into renal or hepatic cysts. This might represent another reason why PLD was more affected by PWV. PWV reflects the continuous intra-vessel pressure, which may promote arterial extension and growth (S3 Fig.). In fact, both the hepatic and renal arteries of patients with ADPKD are highly developed (S4, S5 Fig.). We have reported that the enlargement of TKV might be faster in patients with ADPKD and larger renal arteries [41]. We hypothesized that renal or hepatic arterial growth and an increase in blood flow are essential for the progression of PKD or PLD. Thus, PWV may represent a surrogate maker for such arterial growth. Brain and aortic aneurysms are more prevalent in patients with ADPKD than in the general population [42,43], and the mechanism of the arterial expansion may be similar to those of the renal or hepatic arteries in patients with ADPKD.

The results of the present study suggest that arterial stiffness is associated with increases in LV, and therefore it may be necessary to control hypertension, salt intake, and hyperlipidemia to limit the progression of PLD as well as PKD. Strategies aimed at reducing PWV is important means of preventing such increases in LV, because the LV of patients with low baPWV was unlikely to increase in the present study (Fig 6). The monitoring of baPWV in patients with ADPKD from an early stage and the maintenance of a low baPWV may be important. It may also be necessary to avoid volume overload to limit the progression of PLD, because the vasopressin V2 receptor is unique in renal cyst epithelial cells, and high-water intake may promote the growth of hepatic cysts. In general, high-water intake is recommended for patients with ADPKD. However, physicians should carefully monitor the water balance of patients with ADPKD to avoid volume overload.

Tolvaptan use was found to be a significant determinant of baseline LV and the changes in LV in the present study. One of the reasons for this was that patients taking tolvaptan were likely to have smaller LV and larger TKV, because tolvaptan is more likely to be prescribed for patients with larger TKV. However, as we have reported previously, tolvaptan may be an effective means of limiting LV, especially in patients showing rapid progression of PLD [44]. However, more research is needed on this topic.

The effects of long-term changes in PWV on the increases in LV may be more important, and it will be necessary to conduct a prospective study to clarify the relationship between these parameters. We also need to conduct studies to clarify the relationships of each factor associated with baPWV (hypertension, salt intake, obesity, hyperlipidemia, etc.) with the progression of PLD and the effectiveness of rigorous blood pressure control in patients with PLD. It is also necessary to clarify the relationship between body water volume and the progression of PLD. Finally, we need to evaluate the relationship between cfPWV and LV, because cfPWV is more commonly measured in western countries than baPWV.

The present study had several limitations. First, it was a retrospective study conducted in two hospitals. Second, all the participants were of Asian ethnicity, which may reduce the generalizability of the findings. Third, we only included patients who agreed to undergo baPWV measurement, which may have introduced selection bias. Lastly, not all the participants underwent genetic testing, because this is not usually available in Japan. The study also had some strengths. baPWV is widely recognized to be a reliable clinical measure of arterial stiffness. The number of participants was not small, and the observation period was long. All of the participants underwent abdominal imaging more than once, meaning that we could analyze the longitudinal effects of PWV on kidney and liver volume. Furthermore, the kidney and liver volumes were measured by an independent single group of research assistants using specific, validated software, which minimized measurement error. Statistical analysis was conducted by an independent statistician.

In conclusion, we have shown that baPWV is a predictor of the baseline and sequential changes in LV. It may be important to maintain a low PWV to prevent the progression of PLD in patients with ADPKD. However, further research is needed regarding the value of PWV measurement and the mechanisms of the links of hepatic cyst expansion with PWV.

## Supporting information

S1 Fig Correlation between log (height-adjusted total kidney volume) and eGFR. (TIF)

S2 Fig Graph of the mean height-adjusted total kidney volume ratio against time, created using a generalized additive model, stratified according to ΔeGFR quartile. (TIF)

S3 Fig Mechanism of renal or hepatic artery growth in patients with autosomal dominant polycystic kidney disease. (TIF)

S4 Fig Hepatic arteriography of patients with autosomal dominant polycystic kidney disease. (TIF)

S5 Fig Renal arteriography of patients with autosomal dominant polycystic kidney disease. (TIF)

S1 Table Diagnostic criteria for autosomal dominant polycystic kidney disease proposed by Progressive Renal Disease Research (Ministry of Health, Labour and Welfare of Japan), presented in clinical practice guidelines for autosomal dominant polycystic kidney disease (2nd edition). (DOCX)

S2 Table Clinical characteristics of all enrolled patients at each hospital. (DOCX)
**S3 Table.** **Univariable and multivariable regression coefficient for height-adjusted total kidney volume in patients with ADPKD at** **baseline.**

S4 Table The changes (95% CIs) of slope coefficients of height-adjusted total kidney volume curves by predictive variables in univariable and multivariable linear mixed model analyses. (DOC)

S5 Table A) The changes (95% CIs) of slope coefficients of height-adjusted liver volume curves by predictive variables in univariable and multivariable linear mixed model analyses in female patients. B) The changes (95% CIs) of slope coefficients of height-adjusted liver volume curves by predictive variables in univariable and multivariable linear mixed model analyses in male patients.
(DOC)

S6 Table A) The changes (95% CIs) of slope coefficients of height-adjusted liver volume curves by predictive variables in patients with liver volume ≥1452.5 mL using univariable and multivariable linear mixed model analyses. B) The changes (95% CIs) of slope coefficients of height-adjusted liver volume curves by predictive variables in patients with liver volume <1452.5 mL using univariable and multivariable linear mixed model analyses.
(DOC)

S7 Table A) The changes (95% CIs) of slope coefficients of height-adjusted liver volume curves by predictive variables in patients of age ≥ 48 years using univariable and multivariable linear mixed model analyses. B) The changes (95% CIs) of slope coefficients of height-adjusted liver volume curves by predictive variables in patients of age < 48 years using univariable and multivariable linear mixed model analyses.
(DOC)
