# Supplementary figures and images for: Brachial-ankle pulse wave velocity predicts liver volume in patients with autosomal dominant polycystic kidney disease
Source: PLoS One. 2025 Jul 21;20(7):e0328133. doi: 10.1371/journal.pone.0328133 (PMC12279127; doi:10.1371/journal.pone.0328133)

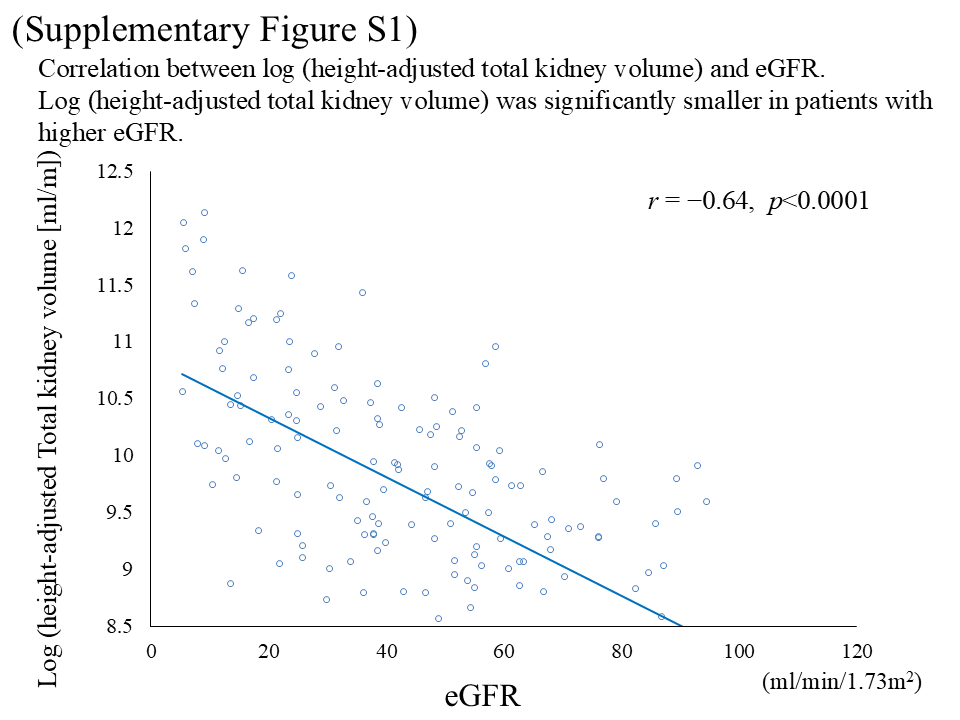

Supplement: S1 Fig — (TIF) [file pone.0328133.s001.tif]

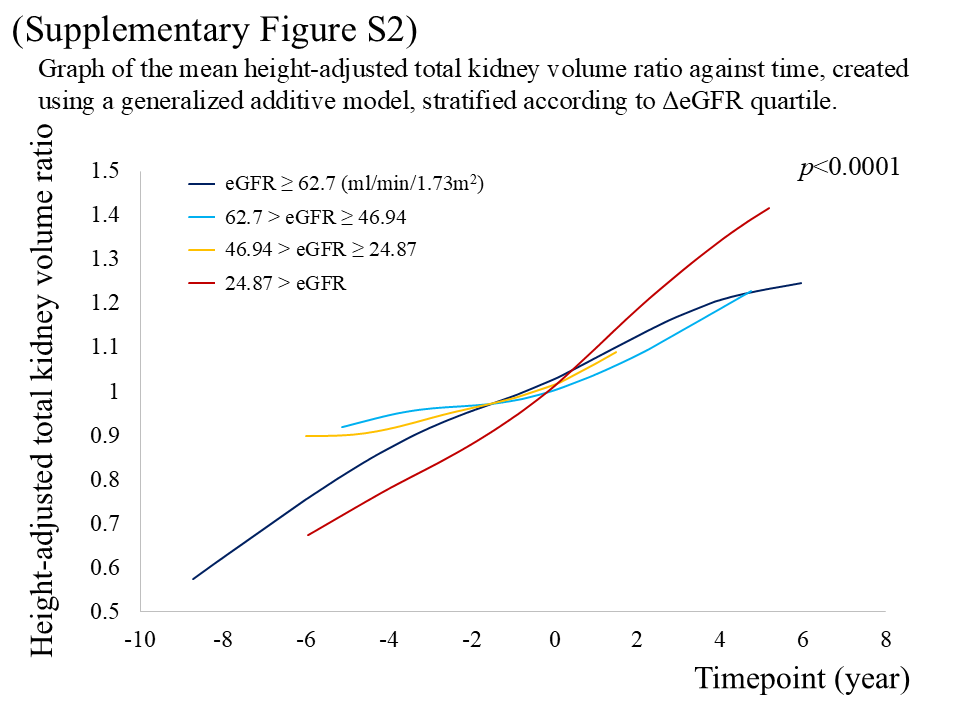

Supplement: S2 Fig — (TIF) [file pone.0328133.s002.tif]

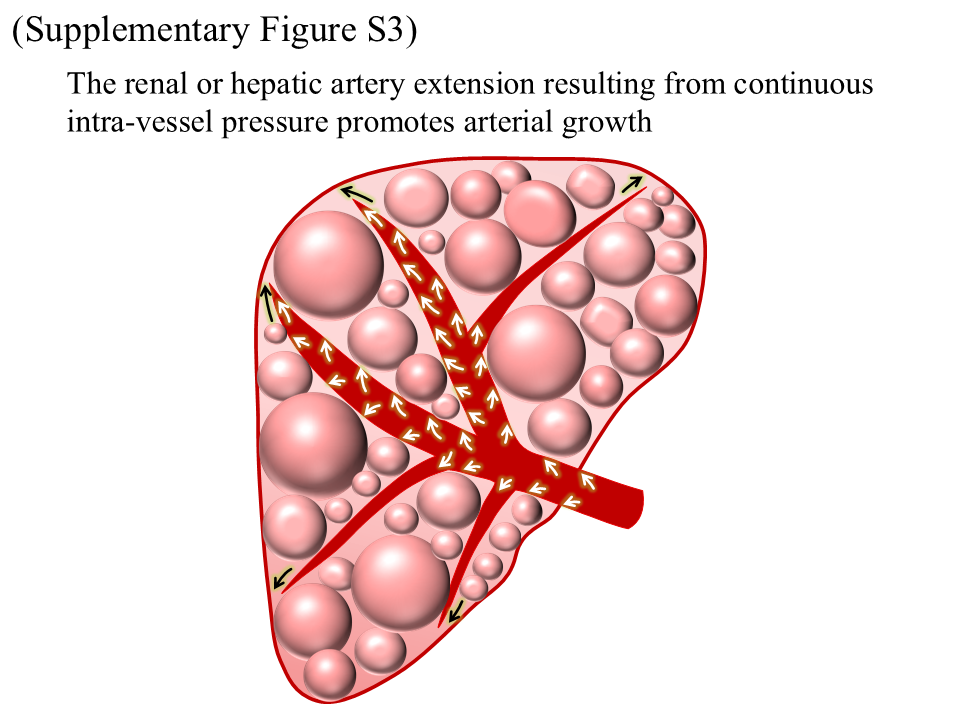

Supplement: S3 Fig — (TIF) [file pone.0328133.s003.tif]

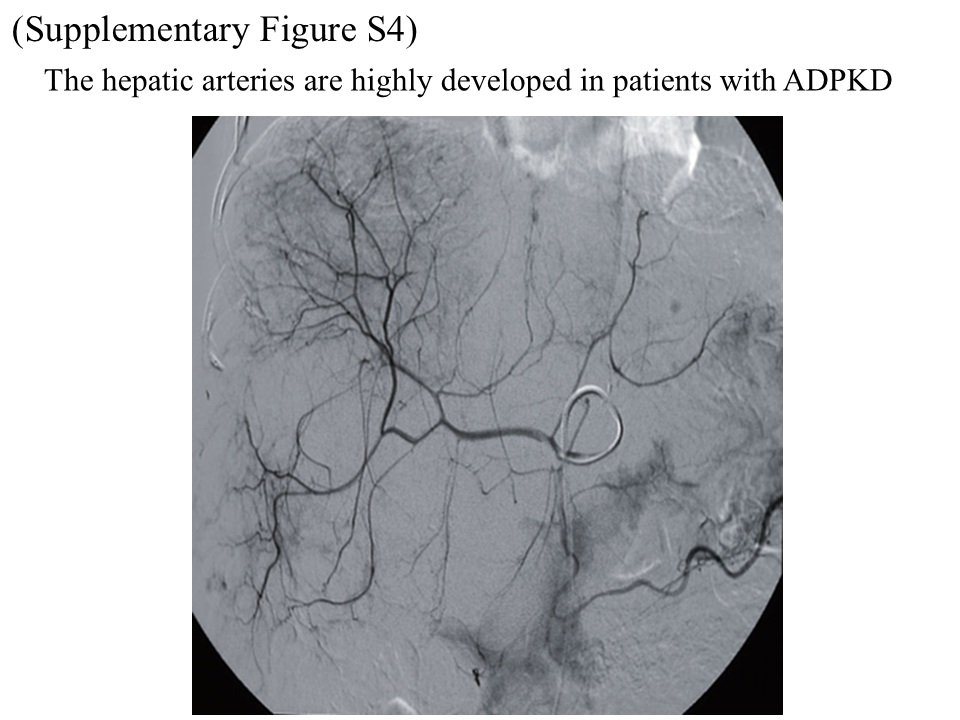

Supplement: S4 Fig — (TIF) [file pone.0328133.s004.tif]

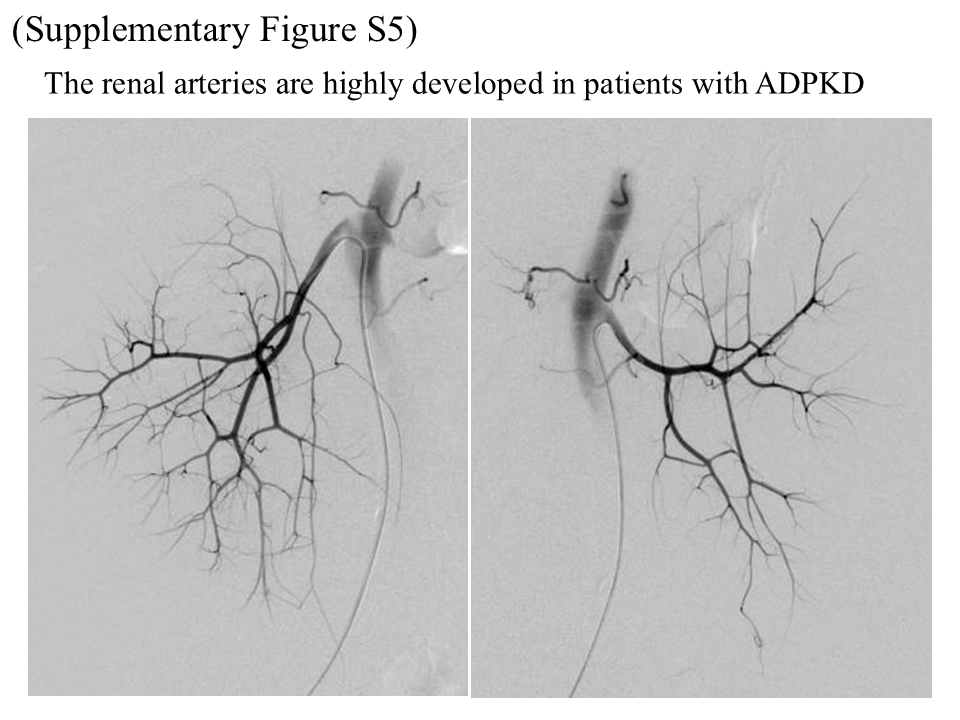

Supplement: S5 Fig — (TIF) [file pone.0328133.s005.tif]
