# Supplementary material for: Brachial-ankle pulse wave velocity predicts liver volume in patients with autosomal dominant polycystic kidney disease
Source: PLoS One. 2025 Jul 21;20(7):e0328133. doi: 10.1371/journal.pone.0328133 (PMC12279127; doi:10.1371/journal.pone.0328133)
Supplement: S1 Table — (DOCX) [file pone.0328133.s006.docx]

**Brachial-ankle pulse wave velocity predicts kidney and liver volume in patients with autosomal dominant polycystic kidney disease**

**Supporting Information**

**(Supplementary Table S1)** Diagnostic criteria for autosomal dominant polycystic kidney disease proposed by Progressive Renal Disease Research (Ministry of Health, Labour and Welfare of Japan), presented in clinical practice guidelines for autosomal dominant polycystic kidney disease (2nd edition).

For individuals with a family history

1. At least three cysts in both kidneys detected by ultrasound.
2. At least five cysts in both kidneys detected by CT or MRI.

For individuals without a family history

1) At least three cysts in both kidneys detected by ultrasound, CT, or MRI in individuals aged 15 years or younger, in addition to exclusion of the diseases listed below.

2) At least five cysts in both kidneys detected by ultrasound, CT or MRI in individuals aged 16 years or older, in addition to exclusion of the diseases listed below.

Diseases to be excluded

multiple simple renal cysts, renal tubular acidosis, multicystic kidney (multicystic dysplastic kidney), multilocular cysts of the kidney, medullary cystic disease of the kidney (juvenile nephronophthisis), acquired cystic disease of the kidney, and autosomal recessive polycystic kidney disease
