# Supplementary material for: Brachial-ankle pulse wave velocity predicts liver volume in patients with autosomal dominant polycystic kidney disease
Source: PLoS One. 2025 Jul 21;20(7):e0328133. doi: 10.1371/journal.pone.0328133 (PMC12279127; doi:10.1371/journal.pone.0328133)
Supplement: S2 Table — (DOCX) S3 Table. Univariable and multivariable regression coefficient for height-adjusted total kidney volume in patients with ADPKD at baseline. [file pone.0328133.s007.docx]

**Brachial-ankle pulse wave velocity predicts kidney and liver volume in patients with autosomal dominant polycystic kidney disease**

**Supporting Information**

**(Supplementary Table S2) Clinical characteristics of all enrolled patients at each hospital**

|  | All patients | Toranomon Hospital Kajigaya | Toranomon Hospital | ^a^ P value |
| --- | --- | --- | --- | --- |
| Number (M/F) | 165 (66 / 99) | 110 (42 / 68) | 55 (24 / 31) | 0.5011 |
| Age (years) [mean±SD] | 47.3 ± 6.9 | 47.2 ± 7.0 | 47.6 ± 6.8 | 0.6921 |
| Height (cm) [mean±SD] | 165.9 ± 8.8 | 165.3 ± 8.6 | 167.2 ± 9.4 | 0.1918 |
| Body weight (kg) [mean±SD] | 64.2 ± 15.1 | 63.7 ± 14.6 | 65.5 ± 16.1 | 0.4660 |
| BMI [mean±SD] | 23.1 ± 3.9 | 23.1 ± 3.9 | 23.2 ± 4.2 | 0.8740 |
| Systolic blood pressure (mmHg) [mean±SD] | 126.0 ± 15.9 | 124.6 ± 14.8 | 129.6 ± 18.1 | 0.1388 |
| Diastolic blood pressure (mmHg) [mean±SD] | 81.7±12.0 | 80.0 ± 11.0 | 85.9 ± 13.6 | 0.0176 |
| Heart rate (per min) [mean±SD] | 70.2 ± 11.9 | 70.4 ± 11.8 | 69.8 ± 12.7 | 0.8309 |
| Right ABI [mean±SD] | 1.14 ± 0.07 | 1.14 ± 0.07 | 1.15 ± 0.08 | 0.5057 |
| Left ABI [mean±SD] | 1.13 ± 0.07 | 1.14 ± 0.07 | 1.14 ± 0.01 | 0.4991 |
| Right baPWV [mean±SD] | 1421.2 ± 225.7 | 1441.1 ± 235.5 | 1381.6 ± 200.9 | 0.1106 |
| Left baPWV [mean±SD] | 1438.8 ± 244.7 | 1464.7 ± 258.5 | 1387.1 ± 207.3 | 0.0546 |
| Mean baPWV [mean±SD] | 1430.0 ± 233.7 | 1452.9 ± 245.4 | 1384.3 ± 202.9 | 0.0757 |
| ΔbaPWV [mean±SD] | 253.3 ± 230 | 279.3 ± 233.8 | 201.4 ± 215.2 | 0.0398 |
| Smoking history [n, (%)] | 47 (28.5 %) | 32 (29.1 %) | 15 (25.7 %) | 0.8379 |
| Medications |  |  |  |  |
| Tolvaptan | 88 (53.3 %) | 51 (46.4 %) | 37 (67.3 %) | 0.0105 |
| Past medical history |  |  |  |  |
| Cardiovascular disease | 7 (4.2 %) | 5 (4.5 %) | 2 (3.6 %) | 0.7821 |
| Cerebral vascular disease | 5 (3.0 %) | 3 (2.7 %) | 2 (3.6 %) | 0.7515 |
| Cerebral aneurysm | 18 (10.9 %) | 12 (10.9 %) | 6 (10.9 %) | 1.0000 |
| Subarachnoid hemorrhage | 6 (3.6 %) | 4 (3.6 %) | 2 (3.6 %) | 1.0000 |
| Sleep Apnea Syndrome | 8 (4.8 %) | 7 (6.4 %) | 1 (1.8 %) | 0.1653 |
| Malignant neoplasm | 5 (3.0 %) | 4 (3.6 %) | 1 (1.8 %) | 0.5026 |
| Diabetes mellitus | 3 (1.8 %) | 3 (2.7 %) | 0 (0.0 %) | 0.1167 |
| Hypertension | 125 (75.8 %) | 81 (73.6 %) | 44 (80.0 %) | 0.3631 |
| Hyperlipidemia | 25 (15.1 %) | 15 (13.6 %) | 10 (18.2 %) | 0.4481 |
| Hyperuricemia | 58 (35.1 %) | 37 (33.6 %) | 22 (40.0 %) | 0.4232 |
| Renal or Liver cyst infection | 9 (5.4 %) | 11 (10.0 %) | 4 (7.2 %) | 0.5589 |
| Laboratory data |  |  |  |  |
| Hb (g/dL) [mean±SD] | 12.9 ± 1.5 | 12.8 ± 1.6 | 12.9 ± 1.4 | 0.7728 |
| Cr (mg/dL) [median (IQR)] | 1.2 (0.8, 1.9) | 1.2 (0.8, 2.1) | 1.2 (0.8, 1.7) | 0.4573 |
| eGFR (ml/min/1.73m^2^) [mean±SD] | 46.1 ± 24.0 | 45.2 ± 25.1 | 47.8 ± 21.8 | 0.5093 |
| Proteinuria (g/gCr) [median (IQR)] | 0.13 (0.06, 0.25) | 0.15 (0.06, 0.29) | 0.08 (0.05, 0.17) |  |
| Image findings |  |  |  |  |
| Hight-TKV (mL) [median (IQR)] | 795 (520, 1271) | 853 (519, 1349) | 724 (515, 1191) | 0.2087 |
| Hight-LV (mL) [median (IQR)] | 1453 (934, 3664) | 2278 (978, 4174) | 1097 (816, 2241) | 0.0020 |
| Observational periods (months) [median (IQR)] | 48.2 (28.5, 55.9) | 48.0 (27.0, 57.2) | 48.2 (32.3, 55.4) | 0.9078 |

SD, standard deviation; IQR, interquartile range (25% - 75%); BMI, body mass index; ABI, ankle brachial pressure Index; baPWV, brachial-ankle pulse wave velocity; ΔbaPWV, baPWV of each participant – the mean value for controls of the same age and sex; eGFR, estimated glomerular filtration rate; TKV, total kidney volume; LV, liver volume

^a^ Differences between four groups were assessed by analysis of variance for continuous variables with a normal distribution and the Kruskal-Wallis test for continuous variables with a non-normal distribution. Differences of categorical variables between groups were assessed by the χ^2^ test or Fisher’s exact test.
