# Supplementary material for: Brachial-ankle pulse wave velocity predicts liver volume in patients with autosomal dominant polycystic kidney disease
Source: PLoS One. 2025 Jul 21;20(7):e0328133. doi: 10.1371/journal.pone.0328133 (PMC12279127; doi:10.1371/journal.pone.0328133)
Supplement: S4 Table — (DOC) [file pone.0328133.s008.doc]

**Brachial-ankle pulse wave velocity predicts kidney and liver volume in patients with autosomal dominant polycystic kidney disease**

**Supporting Information**

**(Supplementary Table S3) Univariable and multivariable regression coefficient for height-adjusted total kidney volume in patients with ADPKD at baseline**

|  | Univariable analysis | | | |  | Multivariable analysis ^a^ | | | |
| --- | --- | --- | --- | --- | --- | --- | --- | --- | --- |
|  | Regression coefficient | 95% CI | | P value |  | Regression coefficient | 95% CI | | P value |
| Sex (Male) | 0.4524 | 0.2495 | 0.6553 | <.0001 |  | 0.3669 | 0.1236 | 0.6102 | 0.0034 |
| Age (per 1 year) | -0.0009 | -0.0161 | 0.0144 | 0.9084 |  | -0.0215 | -0.0387 | -0.0043 | 0.0149 |
| BMI (per 1) | 0.0408 | 0.0150 | 0.0666 | 0.0021 |  |  |  |  |  |
| Systolic BP (per 1 mmHg) | 0.0102 | 0.0021 | 0.0184 | 0.0141 |  |  |  |  |  |
| Diastolic BP (per 1 mmHg) | 0.0158 | 0.0052 | 0.0265 | 0.0039 |  |  |  |  |  |
| Heart rate (per 1) | 0.0021 | -0.0108 | 0.0150 | 0.7456 |  |  |  |  |  |
| Mean baPWV (per 1) | 0.0003 | -0.0002 | 0.0008 | 0.1843 |  |  |  |  |  |
| ΔbaPWV (per 1) | 0.0001 | -0.0003 | 0.0006 | 0.5779 |  |  |  |  |  |
| Smoking history | 0.2271 | -0.0064 | 0.4606 | 0.0565 |  | -0.0277 | -0.2654 | 0.2099 | 0.8181 |
| Tolvaptan | 0.3622 | 0.1590 | 0.5654 | 0.0006 |  |  |  |  |  |
| Cardiovascular disease | -0.0475 | -0.5691 | 0.4742 | 0.8576 |  |  |  |  |  |
| Cerebral vascular disease | 0.4833 | -0.1255 | 1.0921 | 0.1189 |  |  |  |  |  |
| Cerebral aneurysm | 0.2872 | -0.0471 | 0.6215 | 0.0918 |  |  |  |  |  |
| Subarachnoid hemorrhage | 0.4375 | -0.1201 | 0.9951 | 0.1233 |  |  |  |  |  |
| Sleep Apnea Syndrome | 0.2139 | -0.2745 | 0.7023 | 0.3884 |  | -0.3006 | -0.8357 | 0.2344 | 0.2687 |
| Malignant neoplasm | 0.0323 | -0.5811 | 0.6456 | 0.9174 |  |  |  |  |  |
| Diabetes mellitus | 0.4980 | -0.2852 | 1.2812 | 0.2111 |  |  |  |  |  |
| Hypertension | 0.5378 | 0.3070 | 0.7686 | <.0001 |  |  |  |  |  |
| Hyperlipidemia | 0.0989 | -0.1939 | 0.3918 | 0.5057 |  |  |  |  |  |
| Hyperuricemia | 0.5762 | 0.3757 | 0.7767 | <.0001 |  |  |  |  |  |
| Renal or Liver cyst infection | 0.1125 | -0.0023 | 0.2274 | 0.0547 |  |  |  |  |  |
| Hb (per 1 g/dL) | -0.0154 | -0.0840 | 0.0531 | 0.6571 |  |  |  |  |  |
| eGFR (per 1 ml/min/1.73m^2^) | -0.0181 | -0.0215 | -0.0147 | <.0001 |  | -0.0244 | -0.0299 | -0.0189 | <.0001 |
| Log (Proteinuria [g/gCr]) | 0.2328 | 0.1498 | 0.3159 | <.0001 |  | 0.2353 | 0.0086 | 0.4619 | 0.0420 |
| Log (htTLV[mL]) | -0.1660 | -0.3002 | -0.0317 | 0.0157 |  |  |  |  |  |

BMI, body mass index; baPWV, brachial-ankle pulse wave velocity; ΔbaPWV, baPWV of each participant – the mean value for controls of the same age and sex; eGFR, estimated glomerular filtration rate; htTLV, height-adjusted total liver volume

^a^ These variables were selected by stepwise elimination.
