# Supplementary material for: Brachial-ankle pulse wave velocity predicts liver volume in patients with autosomal dominant polycystic kidney disease
Source: PLoS One. 2025 Jul 21;20(7):e0328133. doi: 10.1371/journal.pone.0328133 (PMC12279127; doi:10.1371/journal.pone.0328133)
Supplement: S5 Table — B) The changes (95% CIs) of slope coefficients of height-adjusted liver volume curves by predictive variables in univariable and multivariable linear mixed model analyses in male patients. (DOC) [file pone.0328133.s009.doc]

**Brachial-ankle pulse wave velocity predicts kidney and liver volume in patients with autosomal dominant polycystic kidney disease**

**Supporting Information**

**(Supplementary Table S4)** **The changes (95% CIs) of slope coefficients of height-adjusted total kidney volume curves by predictive variables in univariable and multivariable linear mixed model analyses**

|  | Univariable analysis | | | |  | Multivariable analysis ^a^ | | | |
| --- | --- | --- | --- | --- | --- | --- | --- | --- | --- |
|  | Regression coefficient | 95% CI | | P value |  | Regression coefficient | 95% CI | | P value |
| Sex (Male) | 465.75 | 229.06 | 702.44 | 0.0001 |  | 332.29 | 99.16 | 565.42 | 0.0056 |
| Age (per 1 year) | 4.86 | -12.69 | 22.41 | 0.5852 |  | -13.50 | -29.38 | 2.38 | 0.0948 |
| BMI (per 1) | 40.69 | 10.73 | 70.65 | 0.0081 |  |  |  |  |  |
| Systolic BP (per 1 mmHg) | 13.61 | 4.85 | 22.37 | 0.0026 |  |  |  |  |  |
| Diastolic BP (per 1 mmHg) | 20.39 | 8.95 | 31.83 | 0.0006 |  | 9.70 | 0.81 | 18.58 | 0.0328 |
| Heart rate (per 1) | 2.83 | -11.41 | 17.07 | 0.6940 |  |  |  |  |  |
| Mean baPWV (per 1) | 0.30 | -0.21 | 0.82 | 0.2494 |  |  |  |  |  |
| ΔbaPWV (per 1) | 0.07 | -0.46 | 0.60 | 0.7950 |  |  |  |  |  |
| Smoking history | 346.76 | 79.95 | 613.58 | 0.0112 |  |  |  |  |  |
| Tolvaptan | 240.89 | 0.82 | 480.97 | 0.0492 |  |  |  |  |  |
| Cardiovascular disease | -209.11 | -809.60 | 391.37 | 0.4927 |  |  |  |  |  |
| Cerebral vascular disease | 622.07 | -78.43 | 1322.56 | 0.0814 |  |  |  |  |  |
| Cerebral aneurysm | 339.27 | -45.96 | 724.51 | 0.0839 |  |  |  |  |  |
| Subarachnoid hemorrhage | 410.22 | -234.15 | 1054.60 | 0.2105 |  |  |  |  |  |
| Sleep Apnea Syndrome | 156.92 | -406.87 | 720.70 | 0.5834 |  | -506.36 | -1076.21 | 63.49 | 0.0810 |
| Malignant neoplasm | -142.12 | -848.85 | 564.60 | 0.6918 |  |  |  |  |  |
| Diabetes mellitus | 310.68 | -595.22 | 1216.57 | 0.4992 |  |  |  |  |  |
| Hypertension | 539.50 | 269.26 | 809.74 | 0.0001 |  |  |  |  |  |
| Hyperlipidemia | -23.10 | -361.12 | 314.93 | 0.8928 |  |  |  |  |  |
| Hyperuricemia | 661.85 | 430.60 | 893.09 | <.0001 |  |  |  |  |  |
| Renal or Liver cyst infection | 142.97 | 10.90 | 275.05 | 0.0340 |  |  |  |  |  |
| Hb (per 1 g/dL) | -24.50 | -103.49 | 54.48 | 0.5410 |  |  |  |  |  |
| eGFR (per 1 ml/min/1.73m^2^) | -19.33 | -23.42 | -15.24 | <.0001 |  | -13.88 | -18.53 | -9.24 | <.0001 |
| Log (Proteinuria [g/gCr]) | 220.83 | 157.11 | 284.55 | <.0001 |  | 113.32 | 43.06 | 183.58 | 0.0018 |
| Log (htTLV[mL]) | -103.93 | -211.97 | 4.12 | 0.0593 |  |  |  |  |  |

BMI, body mass index; baPWV, brachial-ankle pulse wave velocity; ΔbaPWV, baPWV of each participant – the mean value for controls of the same age and sex; eGFR, estimated glomerular filtration rate; htTLV, height-adjusted total liver volume

^a^ These variables were selected by stepwise elimination.
