# Supplementary material for: Brachial-ankle pulse wave velocity predicts liver volume in patients with autosomal dominant polycystic kidney disease
Source: PLoS One. 2025 Jul 21;20(7):e0328133. doi: 10.1371/journal.pone.0328133 (PMC12279127; doi:10.1371/journal.pone.0328133)
Supplement: S6 Table — B) The changes (95% CIs) of slope coefficients of height-adjusted liver volume curves by predictive variables in patients with liver volume <1452.5 mL using univariable and multivariable linear mixed model analyses. (DOC) [file pone.0328133.s010.doc]

**Brachial-ankle pulse wave velocity predicts kidney and liver volume in patients with autosomal dominant polycystic kidney disease**

**Supporting Information**

**(Supplementary Table S5A) The changes (95% CIs) of slope coefficients of height-adjusted liver volume curves by predictive variables in univariable and multivariable linear mixed model analyses in female patients**

|  | Univariable analysis | | | |  | Multivariable analysis ^a^ | | | |
| --- | --- | --- | --- | --- | --- | --- | --- | --- | --- |
|  | Regression coefficient | 95% CI | | P value |  | Regression coefficient | 95% CI | | P value |
| Age (per 1 year) | 35.54 | -9.73 | 80.81 | 0.124 |  |  |  |  |  |
| BMI (per 1) | 35.66 | -47.60 | 118.91 | 0.401 |  |  |  |  |  |
| Systolic BP (per 1 mmHg) | -4.03 | -32.14 | 24.08 | 0.778 |  |  |  |  |  |
| Diastolic BP (per 1 mmHg) | 3.38 | -28.61 | 35.37 | 0.836 |  |  |  |  |  |
| Heart rate (per 1) | -28.60 | -8.604 | 28.84 | 0.329 |  |  |  |  |  |
| Mean baPWV (per 1) | 1.26 | 0.13 | 2.40 | 0.028 |  |  |  |  |  |
| ΔbaPWV (per 1) | 1.35 | 0.30 | 2.40 | 0.012 |  | 1.02 | 0.04 | 2.00 | 0.042 |
| Smoking history | -543.31 | -1241.97 | 155.34 | 0.127 |  |  |  |  |  |
| Tolvaptan | -506.61 | -1153.02 | 139.80 | 0.125 |  |  |  |  |  |
| Cardiovascular disease | 1577.80 | 333.25 | 2822.30 | 0.013 |  | 1415.91 | 277.96 | 2553.87 | 0.015 |
| Cerebral vascular disease | -365.80 | -2231.61 | 1500.01 | 0.701 |  |  |  |  |  |
| Cerebral aneurysm | -512.30 | -1256.32 | 231.72 | 0.177 |  |  |  |  |  |
| Subarachnoid hemorrhage | -244.40 | -2113.50 | 1624.69 | 0.798 |  |  |  |  |  |
| Sleep Apnea Syndrome | 1669.35 | -137.10 | 3475.79 | 0.070 |  | 1492.10 | -93.07 | 3077.27 | 0.065 |
| Malignant neoplasm | -365.80 | -2231.61 | 1500.01 | 0.701 |  |  |  |  |  |
| Diabetes mellitus | NA | NA | NA | NA |  |  |  |  |  |
| Hypertension | -192.99 | -784.63 | 398.65 | 0.523 |  |  |  |  |  |
| Hyperlipidemia | -216.80 | -939.06 | 505.47 | 0.556 |  |  |  |  |  |
| Hyperuricemia | -80.44 | -706.59 | 545.71 | 0.801 |  |  |  |  |  |
| Renal or Liver cyst infection | -13.54 | -190.80 | 163.73 | 0.881 |  |  |  |  |  |
| Hb (per 1 g/dL) | -39.15 | -329.46 | 251.15 | 0.792 |  |  |  |  |  |
| eGFR (per 1 ml/min/1.73m^2^) | -5.51 | -17.53 | 6.50 | 0.368 |  |  |  |  |  |
| Log (Proteinuria [g/gCr]) | 699.83 | -31.78 | 1431.43 | 0.061 |  |  |  |  |  |
| Log (htTKV[mL]) | -274.57 | -1309.37 | 760.23 | 0.603 |  |  |  |  |  |

N=99. BMI, body mass index; baPWV, brachial-ankle pulse wave velocity; ΔbaPWV, baPWV of each participant – the mean value for controls of the same age and sex; eGFR, estimated glomerular filtration rate; htTLV, height-adjusted total liver volume

^a^ These variables were selected by stepwise elimination.

**(Supplementary Table S5B) The changes (95% CIs) of slope coefficients of height-adjusted liver volume curves by predictive variables in univariable and multivariable linear mixed model analyses in male patients**

|  | Univariable analysis | | | |  | Multivariable analysis ^a^ | | | |
| --- | --- | --- | --- | --- | --- | --- | --- | --- | --- |
|  | Regression coefficient | 95% CI | | P value |  | Regression coefficient | 95% CI | | P value |
| Age (per 1 year) | 16.45 | -16.82 | 49.72 | 0.333 |  |  |  |  |  |
| BMI (per 1) | 82.16 | 14.29 | 150.03 | 0.018 |  | 96.53 | 34.61 | 158.45 | 0.002 |
| Systolic BP (per 1 mmHg) | -12.77 | -29.47 | 3.92 | 0.134 |  |  |  |  |  |
| Diastolic BP (per 1 mmHg) | -17.50 | -38.84 | 3.84 | 0.108 |  |  |  |  |  |
| Heart rate (per 1) | -38.72 | -123.75 | 46.32 | 0.372 |  |  |  |  |  |
| Mean baPWV (per 1) | 0.76 | -0.27 | 1.79 | 0.149 |  |  |  |  |  |
| ΔbaPWV (per 1) | 0.67 | -0.40 | 1.75 | 0.219 |  |  |  |  |  |
| Smoking history | 32.02 | -438.46 | 502.51 | 0.894 |  |  |  |  |  |
| Tolvaptan | -724.55 | -1280.56 | -168.53 | 0.011 |  | -674.45 | -1193.66 | -155.24 | 0.011 |
| Cardiovascular disease | 47.27 | -763.57 | 858.10 | 0.909 |  |  |  |  |  |
| Cerebral vascular disease | -205.77 | -1126.29 | 714.75 | 0.661 |  |  |  |  |  |
| Cerebral aneurysm | 199.01 | -606.27 | 1004.28 | 0.628 |  |  |  |  |  |
| Subarachnoid hemorrhage | -410.56 | -1521.57 | 700.46 | 0.469 |  |  |  |  |  |
| Sleep Apnea Syndrome | -423.05 | -1337.32 | 491.23 | 0.364 |  |  |  |  |  |
| Malignant neoplasm | 385.28 | -1176.96 | 1947.51 | 0.629 |  |  |  |  |  |
| Diabetes mellitus | -247.07 | -1362.04 | 867.90 | 0.664 |  |  |  |  |  |
| Hypertension | 243.76 | -484.28 | 971.80 | 0.512 |  |  |  |  |  |
| Hyperlipidemia | 397.64 | -140.22 | 935.51 | 0.147 |  |  |  |  |  |
| Hyperuricemia | 257.87 | -199.84 | 715.58 | 0.269 |  |  |  |  |  |
| Renal or Liver cyst infection | 99.38 | -1017.42 | 1216.18 | 0.862 |  |  |  |  |  |
| Hb (per 1 g/dL) | -49.89 | -250.47 | 150.68 | 0.626 |  |  |  |  |  |
| eGFR (per 1 ml/min/1.73m^2^) | -8.53 | -20.63 | 3.57 | 0.167 |  |  |  |  |  |
| Log (Proteinuria [g/gCr]) | 406.24 | -52.38 | 864.86 | 0.083 |  | 370.46 | -32.29 | 773.22 | 0.071 |
| Log (htTKV[mL]) | -172.14 | -1065.74 | 721.46 | 0.706 |  |  |  |  |  |

N=66. BMI, body mass index; baPWV, brachial-ankle pulse wave velocity; ΔbaPWV, baPWV of each participant – the mean value for controls of the same age and sex; eGFR, estimated glomerular filtration rate; htTKV, height-adjusted total kidney volume

^a^ These variables were selected by stepwise elimination.
