# Supplementary material for: Brachial-ankle pulse wave velocity predicts liver volume in patients with autosomal dominant polycystic kidney disease
Source: PLoS One. 2025 Jul 21;20(7):e0328133. doi: 10.1371/journal.pone.0328133 (PMC12279127; doi:10.1371/journal.pone.0328133)
Supplement: S7 Table — B) The changes (95% CIs) of slope coefficients of height-adjusted liver volume curves by predictive variables in patients of age < 48 years using univariable and multivariable linear mixed model analyses. (DOC) [file pone.0328133.s011.doc]

**Brachial-ankle pulse wave velocity predicts kidney and liver volume in patients with autosomal dominant polycystic kidney disease**

**Supporting Information**

**(Supplementary Table S6A)** **The changes (95% CIs) of slope coefficients of height-adjusted liver volume curves by predictive variables in patients with liver volume ≥1452.5 mL using univariable and multivariable linear mixed model analyses**

|  | Univariable analysis | | | |  | Multivariable analysis ^a^ | | | |
| --- | --- | --- | --- | --- | --- | --- | --- | --- | --- |
|  | Regression coefficient | 95% CI | | P value |  | Regression coefficient | 95% CI | | P value |
| Sex (Male) | -189.88 | -1107.45 | 727.70 | 0.685 |  |  |  |  |  |
| Age (per 1 year) | 66.57 | -1.92 | 135.05 | 0.057 |  |  |  |  |  |
| BMI (per 1) | 50.34 | -66.78 | 167.46 | 0.400 |  |  |  |  |  |
| Systolic BP (per 1 mmHg) | 1.26 | -24.14 | 25.66 | 0.923 |  |  |  |  |  |
| Diastolic BP (per 1 mmHg) | 13.62 | -18.81 | 46.05 | 0.410 |  |  |  |  |  |
| Heart rate (per 1) | 3.76 | -79.30 | 86.82 | 0.929 |  |  |  |  |  |
| Mean baPWV (per 1) | 1.20 | -0.15 | 2.55 | 0.081 |  |  |  |  |  |
| ΔbaPWV (per 1) | 1.30 | 0.20 | 2.58 | 0.047 |  | 1.08 | -0.06 | 2.22 | 0.064 |
| Smoking history | -657.31 | -1676.34 | 361.73 | 0.206 |  |  |  |  |  |
| Tolvaptan | -1025.40 | -1861.33 | -189.48 | 0.016 |  | -882.26 | -1657.84 | -106.67 | 0.026 |
| Cardiovascular disease | 59.35 | -1234.44 | 1353.14 | 0.928 |  |  |  |  |  |
| Cerebral vascular disease | NA | NA | NA | NA |  |  |  |  |  |
| Cerebral aneurysm | -170.75 | -1713.11 | 1371.61 | 0.828 |  |  |  |  |  |
| Subarachnoid hemorrhage | NA | NA | NA | NA |  |  |  |  |  |
| Sleep Apnea Syndrome | 614.56 | -1505.15 | 2734.26 | 0.570 |  |  |  |  |  |
| Malignant neoplasm | -813.32 | -2913.04 | 1286.39 | 0.448 |  |  |  |  |  |
| Diabetes mellitus | NA | NA | NA | NA |  |  |  |  |  |
| Hypertension | 444.23 | -459.04 | 1347.50 | 0.335 |  |  |  |  |  |
| Hyperlipidemia | 1143.45 | -44.18 | 2331.07 | 0.059 |  |  |  |  |  |
| Hyperuricemia | 230.02 | -670.03 | 1130.08 | 0.616 |  |  |  |  |  |
| Renal or Liver cyst infection | 221.03 | -491.58 | 933.64 | 0.543 |  |  |  |  |  |
| Hb (per 1 g/dL) | -159.27 | -451.67 | 133.12 | 0.286 |  |  |  |  |  |
| eGFR (per 1 ml/min/1.73m^2^) | -9.07 | -28.16 | 10.02 | 0.352 |  |  |  |  |  |
| Log (Proteinuria [g/gCr]) | 2.00 | -3.02 | 7.03 | 0.433 |  |  |  |  |  |
| Log (htTKV[mL]) | -624.54 | -54.82 | 8570.49 | 0.053 |  | -399.20 | -1600.15 | 801.76 | 0.515 |

N=83. BMI, body mass index; baPWV, brachial-ankle pulse wave velocity; ΔbaPWV, baPWV of each participant – the mean value for controls of the same age and sex; eGFR, estimated glomerular filtration rate; htTKV, height-adjusted total kidney volume; NA, not applicable

^a^ These variables were selected by stepwise elimination.

**(Supplementary Table S6B) The changes (95% CIs) of slope coefficients of height-adjusted liver volume curves by predictive variables in patients with liver volume <1452.5 mL using univariable and multivariable linear mixed model analyses**

|  | Univariable analysis | | | |  | Multivariable analysis ^a^ | | | |
| --- | --- | --- | --- | --- | --- | --- | --- | --- | --- |
|  | Regression coefficient | 95% CI | | P value |  | Regression coefficient | 95% CI | | P value |
| Sex (Male) | 110.30 | 23.27 | 197.33 | 0.013 |  | 22.52 | -75.31 | 120.34 | 0.652 |
| Age (per 1 year) | -6.18 | -12.86 | 0.50 | 0.070 |  |  |  |  |  |
| BMI (per 1) | 21.44 | 9.94 | 32.95 | <0.001 |  | 17.72 | 5.39 | 30.05 | 0.005 |
| Systolic BP (per 1 mmHg) | 3.09 | -1.38 | 7.56 | 0.175 |  |  |  |  |  |
| Diastolic BP (per 1 mmHg) | 0.93 | -4.22 | 6.08 | 0.723 |  |  |  |  |  |
| Heart rate (per 1) | -6.55 | -15.98 | 2.88 | 0.173 |  |  |  |  |  |
| Mean baPWV (per 1) | -0.12 | -0.36 | -0.12 | 0.317 |  |  |  |  |  |
| ΔbaPWV (per 1) | -0.12 | -0.36 | 0.12 | 0.311 |  |  |  |  |  |
| Smoking history | 34.63 | -64.27 | 133.53 | 0.493 |  |  |  |  |  |
| Tolvaptan | -11.40 | -134.60 | 111.81 | 0.856 |  |  |  |  |  |
| Cardiovascular disease | 137.72 | -78.70 | 354.15 | 0.212 |  |  |  |  |  |
| Cerebral vascular disease | 86.41 | -104.09 | 276.91 | 0.374 |  |  |  |  |  |
| Cerebral aneurysm | -92.61 | -223.73 | 38.51 | 0.166 |  |  |  |  |  |
| Subarachnoid hemorrhage | -11.80 | -233.57 | 209.97 | 0.917 |  |  |  |  |  |
| Sleep Apnea Syndrome | -124.41 | -341.60 | 92.79 | 0.262 |  |  |  |  |  |
| Malignant neoplasm | 81.20 | -292.83 | 455.23 | 0.670 |  |  |  |  |  |
| Diabetes mellitus | 39.27 | -226.61 | 305.14 | 0.772 |  |  |  |  |  |
| Hypertension | 53.66 | -63.46 | 170.77 | 0.369 |  |  |  |  |  |
| Hyperlipidemia | 57.18 | -50.87 | 165.22 | 0.300 |  |  |  |  |  |
| Hyperuricemia | 51.63 | -37.53 | 140.79 | 0.256 |  |  |  |  |  |
| Renal or Liver cyst infection | -22.73 | -58.34 | 12.89 | 0.211 |  |  |  |  |  |
| Hb (per 1 g/dL) | 53.03 | 13.36 | 92.70 | 0.009 |  | 34.52 | -7.36 | 76.40 | 0.106 |
| eGFR (per 1 ml/min/1.73m^2^) | -0.64 | -2.92 | 1.64 | 0.582 |  |  |  |  |  |
| Log (Proteinuria [g/gCr]) | 16.58 | -95.56 | 128.73 | 0.772 |  |  |  |  |  |
| Log (htTKV[mL]) | -51.35 | -120.53 | 223.23 | 0.558 |  |  |  |  |  |

N=82. BMI, body mass index; baPWV, brachial-ankle pulse wave velocity; ΔbaPWV, baPWV of each participant – the mean value for controls of the same age and sex; eGFR, estimated glomerular filtration rate; htTKV, height-adjusted total kidney volume

^a^ These variables were selected by stepwise elimination.
